# Demographic and Clinical Patterns of Rheumatoid Arthritis in an Emirati Cohort from United Arab Emirates

**DOI:** 10.1155/2019/3057578

**Published:** 2019-09-25

**Authors:** Rajaie Namas, Abhay Joshi, Zarmeena Ali, Jamal Al Saleh, Mohammed Abuzakouk

**Affiliations:** ^1^Division of Rheumatology, Department of Internal Medicine, Cleveland Clinic Abu Dhabi, Abu Dhabi, UAE; ^2^Dubai Hospital, Dubai, UAE

## Abstract

This retrospective cohort study aimed to assess the demographic and clinical characteristics of rheumatoid arthritis (RA) in Emirati patients attending Cleveland Clinic Abu Dhabi, a large tertiary center in the Middle East. In this study, 414 Emirati patients with RA were evaluated over a 3-year period from April 2015 to April 2018. All patients fulfilled the 2010 RA ACR/EULAR criteria and were assessed for demographic and clinical characteristics. The estimated RA prevalence rate in our population cohort was 2.72%. Females showed predominance (80%) with a higher body mass index (31.4 ± 6.61, *P* = 0.0001) compared to males (28.8 ± 6.03, *P* = 0.0001). The most frequent comorbidity observed was dyslipidemia (43.5%) followed by hypertension (37.9%), diabetes mellitus (34.5%), and gastroesophageal reflux disease (33.1%). Xerophthalmia was the most frequent extra-articular manifestation. Rheumatoid factor and anti-citrullinated peptide were detected in 63.3% and 41.5% patients, respectively, while both were present in 33.3% of patients. Methotrexate, adalimumab, and rituximab were the most frequently prescribed disease modifying agents. In this study, we describe disease features that are unique to United Arab Emirates (UAE) patients and demonstrate that RA has a significant disease burden. Our findings highlight the need for a RA national registry to improve the quality of care of these patients in UAE.

## 1. Introduction

Rheumatoid Arthritis (RA) is the most common form of chronic inflammatory arthritis worldwide [1]. Both, genetic and environmental factors influence the risk of RA. In genetically predisposed individuals, a combination of epigenetic modifications and environmental exposures results in a cascade of events inducing synovitis and consequent destructive arthritis [1]. The disease, which eventually leads to deformities and disability, has become a public health concern principally in the Gulf countries, where RA is becoming more recognized. Several RA cohorts report variability in disease characteristics in different populations [2]. Similarly, data on RA patients from the United Arab Emirates are limited. Furthermore, we did not find any reports in literature on RA affecting solely the native Emirati population.

The main objectives of this retrospective study include: (1) to describe the demographic and clinical characteristics of RA affecting Emirati patients in the United Arab Emirates (UAE), (2) to compare our patient cohort with those from other Gulf countries (Bahrain, Kuwait, Oman, Qatar, Saudi Arabia, and Iran), and previous UAE cohorts.

Our study is the first to examine the socio-demographics, clinical, and pharmacological variables in a local homogenous Emirati population attending a large tertiary center in Abu Dhabi between 2015 and 2018. Since there are no population databases or registries in the country, our findings provide important information regarding the understanding of RA in the region.

## 2. Materials and Methods

### 2.1. Subjects

We performed a detailed retrospective chart review of clinical characteristics of all participants diagnosed with RA according to the American College of Rheumatology/European League against Rheumatism (ACR/EULAR) 2010 criteria [3] attending Cleveland Clinic Abu Dhabi between April 2015 and April 2018. Patients were identified using the electronic medical record database at the Cleveland Clinic Abu Dhabi. Search terms for rheumatoid arthritis, inflammatory arthritis, polyarthritis, and seronegative arthritis were used as keywords. A total of 1604 participants were identified initially of 15231 patients who visited the Internal Medicine and Rheumatology Clinics at the center. The inclusion criteria included subjects more than 18 years of age who had at least two visits with the rheumatologist over a six-month period. Exclusion criteria included subjects less than 18 years of age; those missing follow up visits or missing multiple laboratory data; those having other types of inflammatory arthritides including psoriatic arthritis, reactive arthritis, spondyloarthropathies, and inflammatory bowel disease related arthritis. A total number of 512 participants met the inclusion/exclusion criteria; of these 414 Emirati patients form the cohort for the current analysis.

### 2.2. Study Variables

The medical records of all the subjects were reviewed. Demographics, clinical, laboratory, and treatment data were collected. Data on lifestyle habits and comorbidities including body mass index (BMI), cigarette smoking, marital status, dyslipidemia, hypertension, diabetes mellitus (DM), thyroid disease, chronic kidney disease (CKD), coronary artery disease, gastrointestinal reflux disease, iron deficiency anemia (IDA), sickle cell anemia, thalassemia, asthma, chronic obstructive airway disease (COPD), osteoporosis, migraine headache, depression, and malignancies were obtained. Extra-articular manifestations of rheumatoid arthritis were recorded. All patients were subject to ophthalmic evaluation. Laboratory variables including rheumatoid factor (RF), anti-cyclic citrullinated peptide (anti-CCP), erythrocyte sedimentation rate (ESR), C-reactive protein (CRP), antinuclear antibody (ANA), anti-Sjögren syndrome-related antigen A (anti-SSA/Ro) antibodies, anti-Sjögren syndrome type B antigen (anti-SSB/La) antibodies, anti-Smith antibodies, dsDNA, ribonucleoprotein antibodies, C3, and C4 were obtained. RA-related medications including oral corticosteroids, conventional synthetic (DMARDs) (hydroxychloroquine, methotrexate, leflunomide, and sulfasalazine), targeted synthetic DMARDs (tofacitinib), biologic synthetic DMARDs (infliximab, etanercept, adalimumab, certolizumab, golimumab, and rituximab) and other immunosuppressive agents (azathioprine, cyclophosphamide, and mycophenolate mofetil) were documented.

### 2.3. Statistical Analysis

Descriptive statistics (means, percentages, etc.) were used to summarize the characteristics of the cohort. Continuous variables are summarized as the mean ± standard deviation (SD) while skewed continuous variables are summarized with the median and interquartile range. Comparisons of categorical baseline characteristics in the cohort were performed using Pearson's chi-squared test and/or Fisher's exact test. Comparisons of continuous baseline characteristics were performed using Welch's two sample *t*-test or Wilcoxon's rank sum test. Statistical analyses were performed using R 3.2.2 (R Foundation for Statistical Computing, Vienna, Austria).

## 3. Results

### 3.1. Baseline Characteristics

**Figure 1:**
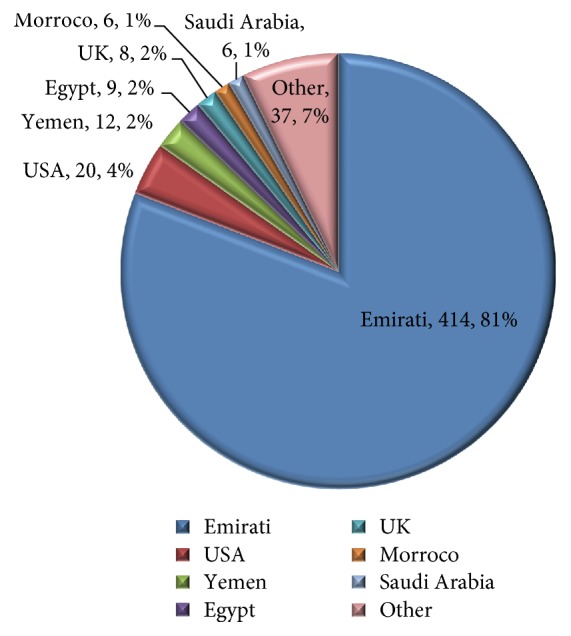
Citizenship of participants (*n*, %).

A total of 512 participants fulfilling the 2010 RA ACR/EULAR criteria were part of this cohort. Among these, 414 participants (81%) were Emirati, while 98 participants (19%) belonged to other nationalities (United States: 4%; Yemen: 2%; Egypt: 2%; United Kingdom: 2%; Morocco: 2%; Saudi Arabia: 1% and others: 37.7%) (Figure 1). Given the paucity of rheumatoid arthritis data in the UAE, we analyzed the findings of the 414 Emirati participants only. The estimated rheumatoid arthritis prevalence rate among patients who visited the Internal Medicine and Rheumatology Clinics at the center in the given time period, based on our analysis was 2.72% (male: 1.28, female: 3.73) (Table 1). Two hundred and ninety one patients (70%) were from Abu Dhabi, 19 (5%) from Dubai. The mean age of female participants was 49.6 ± 13.6 years (mean ± SD) and that of males was 50 ± 15.6 (*P* = 0.6942) ranging from 36 to 65 years. A female preponderance (80%) was observed. There was no statistical difference in disease duration between the two genders, females (6.1 ± 4.6 years) compared to males (5.7 ± 4.9 years, *P* = 0.22). Eighty seven percent of participants fulfilled the 2010 RA ACR/EULAR criteria of ≥6 points.

Thirteen percent of participants had a family history of underlying autoimmune condition with highest percentage being RA (9.2%) followed by psoriasis (1.2%), systemic lupus erythematosus (0.2%), multiple sclerosis (0.5%), scleroderma (0.2%), and ulcerative colitis (0.2%).

### 3.2. Comorbidities and Modifiable Risk Factors

**Figure 2:**
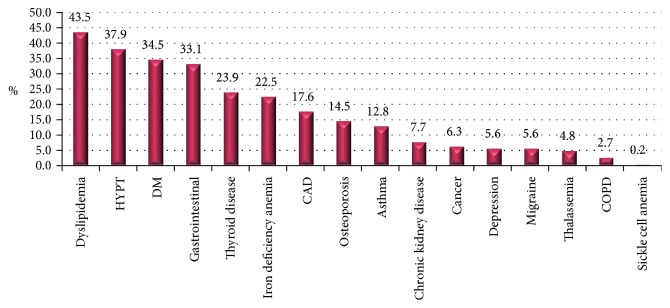
Comorbidities of participants. CAD, coronary artery disease; COPD, chronic obstructive airway disease; DM, diabetes mellitus; HYPT, hypertension.

Forty seven (11%) of 414 participants had a history of past/present smoking. A statistically significant difference was observed in BMI at the time of diagnosis among females and males (*P* = 0.0001) with a higher BMI in females (31.4 ± 6.61) compared to males (28.8 ± 6.03). Five percent of participants underwent a gastric bypass surgery; gastric bypass sleeve being the most frequent (2.9%). The most frequent comorbidity observed was dyslipidemia (43.5%) followed by hypertension (37.9), DM (34.5%) and GERD (33.1%). Other comorbidities observed include thyroid disease (23.9% with hypothyroidism), IDA (22.5%), CAD (17.6%), osteoporosis (14.5%), asthma (12.8%), CKD (7.7%), cancer (6.3%), depression (5.6%), migraine headaches (5.6%), thalassemia (4.8%), COPD (2.7%), and sickle cell anemia (0.2%) (Figure 2).

### 3.3. Extra-Articular Manifestations

**Figure 3:**
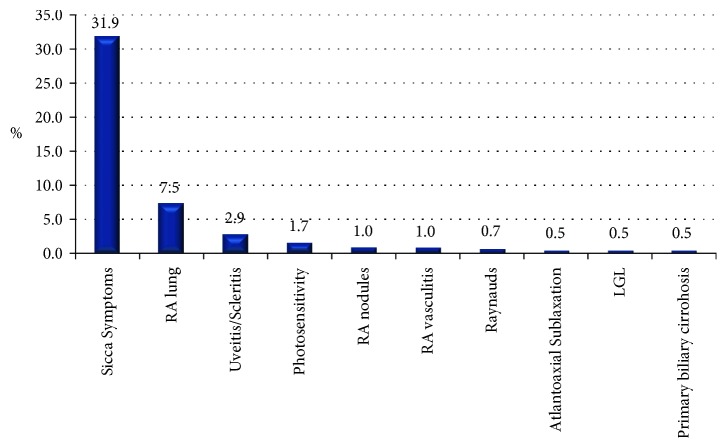
Extra articular manifestations. LGL, T-cell large granular lymphocyte leukemia; RA, rheumatoid arthritis.

Xerophthalmia was the most frequent extra-articular manifestation (31.9%), followed by rheumatoid arthritis associated interstitial lung disease (7.5%), uveitis/scleritis (2.9%), photosensitivity (1.7%), RA nodules (1%), and RA vasculitis (1%) (Table 2). Other less common extra-articular features observed included Raynaud's phenomena (0.7%), atlantoaxial subluxation (0.5%), T-cell large granular lymphocyte leukemia (0.5%), and primary biliary cirrhosis (0.5%) (Figure 3).

### 3.4. Infections

Fourteen percent of the participants who were screened at the time of diagnosis were found to be positive for hepatitis A (12.6%), B (1.2%), C (1%), or latent tuberculosis (12.6%). There were no reported cases of herpes zoster infections.

### 3.5. Malignancy

Twenty six participants (6.3%) had a history of malignancy at the time of diagnosis. Thyroid cancer, lung cancer, and renal cell carcinoma were the most frequent in RA participants (1.9%, 1%, and 1%, respectively). Other types of cancers observed include lymphoma (0.7%), breast cancer (0.5%), leukemia (0.5%), cervical cancer (0.2%), colon cancer (0.2%), ovarian cancer (0.2%), and prostate cancer (0.2%).

### 3.6. Inflammatory Markers, Autoantibody Profile and Radiographic Changes

The mean ESR levels were significantly higher in females (41.5 ± 24.2) compared to males (34.7 ± 24.5, *P* = 0.001). No statistical significance in CRP levels was attained between females and males (*P* = 0.163). Although the DAS scores were calculated as part of our clinical practice, their assessment has not been included in this manuscript as this is a descriptive study. Quantitative analysis using DAS will be considered as part of a separate study with the objective of assessing disease activity at baseline and after 6 months.

**Figure 4:**
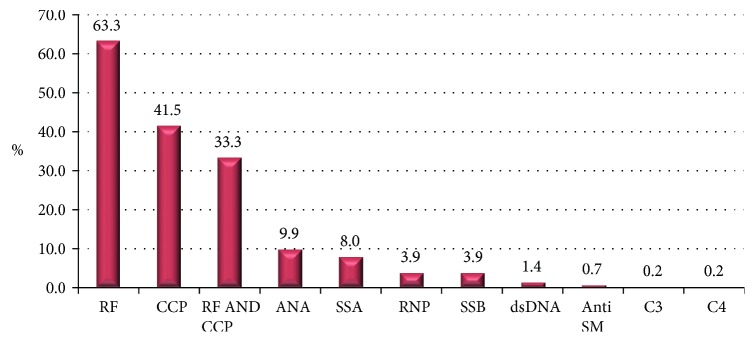
Autoimmune profile of participants. ANA, antinuclear antibody; Anti SM, anti-Smith antibodies; CCP, cyclic citrullinated peptide; dsDNA, double stranded deoxyribonucleic acid; ESR, erythrocyte sedimentation rate; RF, rheumatoid factor; SSA, Sjögren syndrome-related antigen A; SSB, Sjögren syndrome type B antigen.

Autoantibody screens were performed on all 414 patients. Two hundred and sixty two of 414 (63.3%) were rheumatoid factor positive, 172 (41.5%) were anti-CCP positive, and 138 (33.3%) were seropositive for RF and anti-CCP antibody. Other autoantibodies detected include ANA (9.9%), SSA (8%), and SSB (3.9%). The levels of these autoantibodies were low and clinically insignificant (Figure 4). Furthermore, 24.9% of participants had erosive changes on either X-ray or/and magnetic resonance imaging on initial evaluation.

A comparison of seronegative and seropositive patients for RF and anti-CCP antibody was performed with respect to variables such as gender, comorbidity, erosions on X-ray and BMI. The results are provided in Table 3.

### 3.7. Medications

Conventional synthetic DMARDs were the most prescribed medications. Methotrexate being most frequent (62.6%), followed by hydroxychloroquine (36.2%), leflunomide (11.4%), and sulfasalazine (10.1%). Adalimumab was the most frequent subcutaneous biologic synthetic DMARD (16.4%) followed by etanercept (12.6%), infliximab (4.3%), certolizumab pegol (3.6%), and golimumab (2.2%). Rituximab was the most frequent among intravenous biologic synthetic DMARDs (4.3%) followed by abatacept (3.6%) and tocilizumab (3.1%). Thirty one participants (7.4%) were on a targeted synthetic DMARD (tofacitinib citrate). Other immunosuppressive medications used include azathioprine (3.1%) and mycophenolate mofetil (1.4%).

## 4. Discussion

Rheumatoid arthritis is a heterogeneous disease, with variable clinical presentation and characteristics. Data on rheumatoid arthritis in the UAE is very scarce. We report the first comprehensive analysis on a large cohort of Emirati patients attending Cleveland Clinic Abu Dhabi, one of the largest tertiary hospitals in the region. We have analyzed data on 414 participants attending the clinic over a 3-year period (April 2015–April 2018) and have compared our findings with other major cohorts in the Gulf countries. We describe features of RA that are unique to UAE patients, in addition to describing ones that are similar to patients from other Gulf countries.

Data on RA prevalence in solely Emirati population did not exist before our study and based on our analysis, the estimated prevalence of the disease appears to be 2.72%. Higher estimated prevalence was observed in females (3.73%) compared to males (1.28%). In the Middle East and North Africa (MENA) region, the epidemiology of RA is not well identified due to the lack of data on its incidence, prevalence, and disease activity among Arab populations. In our current study we believe that a high prevalence can be due to high consanguinity which can reach up to 50% in some countries raising the question of genetic predisposition as a potential explanatory factor.

The mean age (females: 49.6 ± 13.6 years; males: 50 ± 15.6 years) of the participants observed in the present study was very similar to what was reported in a Kuwaiti cohort (50.6 ± 12 years) [4]. However, a previous study from the UAE showed a younger age of the participants by approximately seven years (42.2 ± 14.3) [5]. This variation could be related to the small number of patients assessed in the previous study and lack of differentiation of Emirati from non-Emirati patients. Similar findings were observed in the Saudi Arabia cohort with age difference from our cohort in the range of 3 to 5 years (46 ± 13) [6].

RA in the current study is substantially more common in females (80%) compared to males (20%), where the female : male ratio is 4 : 1. Female gender was also predominant in previous cohorts from the UAE [5], Iran [7], and Saudi Arabia [8] (Table 1). Female predominance to a lesser extent was observed in cohorts from Kuwait (62.3%). This female predominance is probably related to the effect of endogenous sex hormones, which have complex effects on the immune system [9]. However, the full explanation for why the disease is so uncommon in men remains elusive. Previous literature supports the perception that RA is significantly worse in women when compared with men, pointing to gender-based differences in the course and outcomes of RA [10–12]. The gender difference observed in our cohort has a prognostic value in identifying patients with severe disease as well as aiding in offering tailored and individualized patient treatment and care.

Although RA is one of the most prevalent chronic inflammatory joint diseases, little is known about the magnitude of diagnostic delay in patients followed–up in routine clinical practice. The mean duration of disease at the time of diagnosis in our cohort is 6.1 years, which was similar to that observed in the Kuwait cohort (6.1 years) and Saudi Arabia (5.51 years). Longer durations were observed in the Iranian cohort (7.28 years).

There may be several factors associated with patient behavior and referral systems, contributing to a delay in RA diagnosis and presentation to a specialist rheumatology service. These factors may be related to (1) patients, who due to their belief in herbal remedies, holistic approach, and alternative remedies delay their visit to the specialist, even after experiencing painful and swollen joints, (2) the referring physician, who attributes the joint pain to other conditions such as overuse, degenerative diseases, or gout or (3) the health system and long waiting times to see specialists in rheumatology. Recommendations and guidelines reflect that delay of diagnosis is a major challenge not only in RA but also in other rheumatologic and non-rheumatologic conditions. These challenges need to be addressed in larger studies particularly in the Gulf countries.

Eleven percent of the participants in our study were smokers and this was comparable to what was reported from Kuwait (9.2%, 10.6%). Smoking is well-recognized as an important factor in the etiology and the severity of RA [13]. It is yet to be determined if the smokers in our cohort had more severe and uncontrolled disease.

There are limited data regarding the prevalence of chronic diseases in the general population in the UAE. However, a population-wide cardiovascular screening program among 50,138 adult nationals from the Abu Dhabi emirate of the UAE reported the prevalence of diabetes to be 18%, while dyslipidemia and hypertension prevalence was 44% and 23.1%, respectively [14]. While the figures for dyslipidemia correspond, the prevalence rates for diabetes and hypertension are considerably higher in our cohort. This study has been carried out in a population of patients attending the Internal Medicine and Rheumatology Clinics at the Cleveland Clinic Abu Dhabi. It is therefore already highly selected for various diseases. This could explain the high rates seen in our cohort.

Data on comorbidities in RA patients from Gulf countries is reported in the Kuwait cohort [4]. There was a significant difference in the prevalence of comorbidities among the 2 countries. Diabetes mellitus, hypertension, and bronchial asthma were the most prevalent comorbidities (20.8%, 20.2%, and 11.7%, respectively) reported in the Kuwait cohort; while, dyslipidemia, DM, thyroid disease, and cancer (43.5%, 34.5%, 23.9%, and 6.3%, respectively) were more prevalent in our study compared to the Kuwait cohort (10.5%, 20.8%, 10.4%, and 0.5%, respectively). These differences in the prevalence of comorbidities in the two populations from the same region with similar living conditions and dietary habits can likely be attributed to genetic factors. Furthermore, there was a statistically significant difference in BMI at the time of diagnosis between females and males (*P* = 0.0001) with a higher BMI observed in females. These results correspond with the findings of the National cardiovascular screening program, ‘Weqaya', in which the prevalence rates of obesity in adults aged 30 years and above is higher among females than in males [14]. Emirati women, particularly house wives, tend to be more obese than their male counterparts and this is ascribed to a sedentary lifestyle, multiple pregnancies, and poor diet choices. BMI in the female patients in our study was higher to what was previously reported from the UAE (total: 28.8 ± 6.3, males: 28.2 ± 4.9, females: 28.9 ± 6.6) [15], Kuwait (BMI ≥ 30 in 35–50% of patients) [16]. A small number of our patients (5%) underwent gastric bypass surgery.

Long-term progression of joint damage is best predicted at baseline by multiple factors including the presence of rheumatoid factor, high ESR or CRP level, presence of anti-CCP antibodies, and early radiographic erosions [17, 18]. These predictors were assessed and were found to be present in the current study at baseline indicating the presence of aggressive disease on initial presentation in this population. However, the incidence of bone erosions appears to be lower than that seen in other studies from the UAE and the Kingdom of Saudi Arabia. Bone erosions in our study have been reported based on either X-rays or MRIs. Conventional radiotherapy is shown to be less sensitive for bone erosions, especially in the early stages of the disease. We believe that this could be the reason for the under-reporting of bone erosions in our study. Even though the number of patients is limited in the current study, data suggest that comorbidities are more prevalent in seropositive RA patients compared to seronegative patients. Previous studies have shown that comorbidities are common in patients with RA, with inflammatory activity being the cause of this association [19]. Our study, however, seems to be unique in evaluating comorbidities as a function of patients' seropositive or seronegative immune profile. Further large scale studies are needed to throw light on this association.

Conventional synthetic DMARDs (csDMARDs) were the most prescribed medications in our patient cohort with methotrexate being most frequently used (62%). Methotrexate was used in 62.4% and 65.3% of cases in Iran and Qatar, respectively [7, 8, 10–20]. Leflunomide was not preferred as a first line csDMARD in the UAE (11.4%); this is possibly due to its long term side effects, the necessity of regular blood monitoring and the availability of relatively safe and more effective medications. Other csDMARDs provided include hydroxychloroquine (36.2%), and sulfasalazine (10.1%). All patients have received at least one csDMARD before biologics. More than 50% of the participants were prescribed a biologic synthetic DMARD (bsDMARD), which is higher than what was reported in Qatar (29%) [21] and Kuwait (24%) [22]. Multiple rationales have been postulated for the use of biologics. They are: (1) easy accessibility to health care and multiple recommendations by different rheumatologists, (2) late diagnosis and more aggressive disease at the time of presentation, and (3) patient education and expectations with regard to the disease and the belief that it is curable.

In summary, we report the socio-demographics, clinical, and pharmacological variables in a large cohort of Emirati patients diagnosed with RA and attending a large tertiary center in Abu Dhabi between 2015 and 2018. Since there are no population databases or registries in the country, our findings provide important information regarding the understanding of RA in the region.

The current study is focused on the Emirati population, and completely excludes the expatriate population. The UAE Government provides its nationals/Emiratis with a health care system administered by its Federal Ministry of Health. Cleveland Clinic accepts patients from all the 7 Emirates of UAE, no patient is denied access to care. It may be hypothesized that UAE nationals are associated with a higher economic status that may relate to the comorbidities and incidence of bone erosions.

The major strength of this study is that it is the first to examine a wide range of RA variables in a local homogenous Emirati population and compare the findings with RA patients from other Gulf countries. The study reported the findings from a single large center in Abu Dhabi, therefore the true prevalence of RA in the UAE could not be calculated. Furthermore, comprehensive data regarding the incidence of comorbidities is not available for the local Emirati population making it difficult to determine if comorbidities such as hyperlipidemia or malignancy are truly raised in RA patients.

In conclusion, our study demonstrates that RA is not uncommon in the Emirati population and has a significant disease burden. Our findings highlight the need for a national registry for RA patients to identify the true disease prevalence and improve quality of care of these patients in the UAE.

## Figures and Tables

**Table 1:** Baseline characteristics of participants.

Study		Year	City	Number of participants	Prevalence (%)	Females (%)	F : M	Mean age (years)	Disease duration (years)	BMI kg/m^2^	Smoking (%)	Marital status (%)	Family history of autoimmune disease (%)
Current study		April 2015–April 2018	Abu Dhabi	414	1.62	80	4 : 1	F: 49.6 ± 13.6, M: 50 ± 15.6	F: 6.1 ± 4.6, M: 5.7 ± 4.9	F: 31.4 ± 6.61, M: 28.8 ± 6.03	11	89 married	13
UAE	Badsha et al. [5]	Feb 2006–Dec 2006	Dubai	100	NA	87	5 : 1	42.2 ± 12.3	5.1 ± 5.9	NA	NA	NA	NA
	Al Saleh et al. [15]	Jan 2009–Dec 2009	Dubai	3985	0.90	77	NR	42.1 ± 15.8	NR	28.8 ± 6.3	NR	NR	NR
Saudi Arabia	S. R. Alballa [8]	Jan 1986– Dec 1990	Riyadh	195	NA	78.5	4 : 1	F: 38.6 ± 13.4, M: 42.9±13.3	Median 4	NA	NA	NA	NA
	Suzan M. Attar [23]	Jan 2011–Dec 2013	Jeddah	180	NA	85.60	NA	40.49 ± 12.19	5.51 ± 5.78	NA	NA	NA	NA
	Abdulaziz et al. [6]	Jan 2005–July 2011	Jeddah	139	NA	84.90	NA	46 ± 13	7.24	NA	NA	NA	NA
Kuwait	Al-Awadhi A et al. [24]	2002	Kuwait	359	2.72	89	NA	39 ± 11	10.7 ± 7.6	NR	NR	NR	NR
	Al-Herz et al. [16]	Feb 2013–Dec 2015	Kuwait	952	NA	63.60	NA	54 ± 12	6.3 ± 7.8	NA	10.60	NA	16.9
	Al-Herz et al. [4]	Feb 2013–Dec 2015	Kuwait	835	NA	62.30		50.6 ± 12	6.1 ± 6	NA	9.20	NA	17.1
Oman	Pountain G. [25]	1991	Oman	1925	0.84	NA	NA	NR	NR	NA	NA	NA	NA
	Al- Huder et al. [26]	2015	Oman	30	NA	90	NA	40 ± 13.4	NA	NA	NA	NA	NA
Qatar	Alam et al. [27]	June 2013–Nov 2015	Qatar	496	NA	76	NA	NA	NA	NA	NA	NA	NA
	Emadi et al. [28]	2014	Qatar	294	NA	76	NA	F: 50.8, M: 51.2	NA	NA	NA	NA	NA
	Lutf A. et al. [21]	2013	Qatar	100	NA	67	NR	47 ± 13.4	NA	NR	NR	NR	NR
Iran	Davatchi F. et al. [29]	2014	Tehran	10,291	0.33	85.70	NR	52.3 ± 17.6	NR	NR	NR	74.3 married	NR
	Davatchi F. et al. [30]	2009	Tuyserkan	1,565	0.19	55.10	0.8 : 1	NR	NR	NR	NR	NR	NR
	Sandoughi et al. [7]	Oct 2012–Oct 2013	Zahedan	500	NR	87.40	NR	48.78 ± 13.97	7.28 ± 7.14	NR	13.40	NR	NR

**Table 2:** Clinical and serological features of RA participantsss.

	Current study	UAE [5, 15]	KSA [6, 8, 23, 31, 32]	Kuwait [4]	Oman [25]	Qatar [27]	Iran [7]
*Comorbidities*							
Dyslipidemia %	43.5	NR	55	10.5	NR	59.2	25.8
Hypertension %	37.9	NR	5	20.2	NR	59.2	24.4
Diabetes mellitus %	34.5	NR	4	20.8	NR	8	12
Gastrointestinal %	33.1	NR	NR	2.3	NR	NR	NR
Thyroid disease %	23.9	NR	NR	10.4	NR	NR	9.6
Iron deficiency anemia %	22.5	22	61	NR	NR	NR	NR
Cardiovascular disease %	17.6	NR	3	3.1	NR	NR	NR
Osteoporosis %	14.5	25.80	NR	9.5	NR	NR	NR
Bronchial asthma %	12.8	NR	NR	11.7	NR	NR	NR
Chronic kidney disease %	7.7	NR	6	NR	NR	NR	NR
Cancer %	6.3	NR	3	0.5	NR	NR	NR
Depression %	5.6	NR	NR	NR	NR	NR	NR
Migraine headaches %	5.6	NR	NR	NR	NR	NR	NR
Thalassemia trait %	4.8	NR	NR	NR	NR	NR	NR
COPD %	2.7	NR	NR	NR	NR	NR	NR
Sickle cell trait %	0.2	NR	NR	NR	NR	NR	NR
G6PD deficiency %^†^	4.59	NR	NR	NR	NR	NR	NR

*Autoimmune profile*							
ESR (mean ± SD), mm/h	40.2 ± 24	33 ± 25	**NR**	22.8 ± 19.3, 24.8 ± 16.6	NR	NR	NR
CRP (mean ± SD), mg/L	15.4 ± 21.3	NR	15.19 ± 38.23	NR	NR	NR	NR
Rheumatoid factor %	63.3	73, 59	65, 63, 79.5	75.6	56.7	NR	62.8
Anti-CCP %	41.5	50	57.2, 78.4	57.8	63.3	NR	NR
RF and anti-CCP %	33.3	NR	NR	49	NR	NR	NR
RF (mean)	NR	NR	NR	NR	NR	NR	NR
Anti-CCP (mean)	NR	NR	NR	NR	NR	NR	NR
ANA %	9.9	NR	NR	19.1	NR	NR	NR
SSA %	8	NR	NR	NR	NR	NR	NR
SSB %	3.9	NR	NR	NR	NR	NR	NR
Anti-Smith U/ml	0.7	NR	NR	NR	NR	NR	NR
Anti-RNP %	3.9	NR	NR	NR	NR	NR	NR
dsDNA IU/ml	1.4	NR	NR	NR	NR	NR	NR
Complement 3 g/L	0.2	NR	NR	NR	NR	NR	NR
Complement 4 g/L	0.2	NR	NR	NR	NR	NR	NR
Vitamin D (mean ± SD)	66.8 ± 37.7	NR	NR	NR	NR	NR	NR
Uric acid (mean ± SD)	293.4 ± 37.3	NR	NR	NR	NR	NR	NR

*Infectious workup*							
Hepatitis A %	12.6	NR	NR	NR	NR	NR	NR
Hepatitis B %	1.2	NR	NR	0.47	NR	NR	NR
Hepatitis C %	1	NR	NR	1.19	NR	NR	NR
TB exposure %	12.6	NR	8.4, 6	NR	NR	NR	NR
Shingles %	0	NR	NR	NR	NR	NR	NR
Erosions on imaging %	24.9	55.2, 45.2	79.5	13.6, 8	NR	NR	NR
Extra articular manifestations %	NR	NR	NR	NR	NR	NR	NR
Xerophthalmia %	31.9	28	3, 14.4, 2	36.4, 16.3	NR	NR	NR
RA-ILD %	7.5	NR	11.2, 10	NR	NR	NR	NR
Ocular manifestations %	2.9	NR	NR	NR	NR	NR	NR
Photosensitivity %	1.7	NR	NR	NR	NR	NR	NR
RA nodules %	1	4	13.6, 30, 15.9	3.1	NR	NR	2.6
RA vasculitis %	1	NR	NR	NR	NR	NR	NR
Raynaud's phenomena %	0.7	NR	NR	NR	NR	NR	NR
Atlanto axial subluxation %	0.5	NR	NR	NR	NR	NR	NR
T-LGL %	0.5	NR	NR	NR	NR	NR	NR
Primary biliary cirrhosis %	0.5	NR	NR	NR	NR	NR	NR

^†^Number of patients who had the test done = 88.

RA-ILD, rheumatoid arthritis associated interstitial lung disease; TB, Tuberculosis; T-LGL, T-cell large granular leukemia; RNP, Ribonucleoprotein particle; dsDNA, Deoxyribonucleic acid; SSA, Sjögren syndrome-related antigen A; SSB, Sjögren syndrome-related antigen B; CCP, Cyclic citrullinated peptide; COPD, Chronic obstructive airway disease; G6PD, glucose-6-phosphate dehydrogenase; ESR, Erythrocyte sedimentation rate; CRP, C-reactive protein.

**Table 3:** Comparison of seropositive and seronegative patients for RF and anti-CCP.

Variable	RF or anti-CCP		*P* value
*Gender*	*Seronegative*	*Seropositive*	
Male	31	65	<0.05
Female	123	293	<0.05

*Comorbidity*	*Seronegative*	*Seropositive*	
Diabetes	50	109	<0.05
Hypertension	51	140	<0.05
Thyroid disease	35	85	<0.05
Cancer	7	26	<0.05
Dyslipidemia	63	153	<0.05
CAD	26	69	<0.05
CKD	8	28	<0.05
Osteoporosis	14	55	<0.05
Gastro-intestinal	46	99	<0.05
Iron deficiency anemia	34	77	<0.05
Thalassemia	7	14	>0.05
Sickle cell anemia	0	2	>0.05
G6PD	6	15	>0.05
Asthma	22	37	>0.05
COPD	1	11	<0.05
Migraine	11	14	>0.05
Depression	9	18	>0.05

*Erosion on X-ray*	*Seronegative*	*Seropositive*	
No	107	284	<0.05
Yes	47	74	<0.05

*Weight Class (BMI)*	*Seronegative*	*Seropositive*	
Normal	26	71	<0.05
Obese	79	191	<0.05
Overweight	45	92	<0.05
Underweight	4	4	>0.05

BMI, body mass index; CAD, coronary artery disease; CCP, citrullinated peptide; CKD, chronic kidney disease; COPD, chronic obstructive pulmonary disease; G6PD, glucose-6-phosphate dehydrogenase; RF, rheumatoid factor.

## Data Availability

The cohort data used to support the findings of this study may be released upon application to the Institutional Review Board of Cleveland Clinic Abu Dhabi, who can be contacted at REC@clevelandclinicabudhabi.ae.
